# A teaching coordinator’s nightmare?

**DOI:** 10.3205/zma001256

**Published:** 2019-10-15

**Authors:** Andreas Winkelmann

**Affiliations:** 1 Medizinische Hochschule Brandenburg Theodor Fontane, Institut für Anatomie, Neuruppin, Germany

**Keywords:** standard curriculum, reformed curriculum, teaching coordination, interdisciplinary teaching, modularity

## Abstract

This field report compares the planning and coordination effort of an anatomy teaching coordinator in a subject-oriented standard curriculum with a cross-subject, modularly organized reformed curriculum at a faculty with 600 medical students per year. The distribution of the anatomical teaching over several locations and modules in all semesters, as well as the rotation of these modules within the semester, results in an increased amount of coordination of teaching content and in particular a very complicated timetable. Appropriate and nevertheless non-overlapping allocation of anatomy teaching staff in this timetable is a special challenge. There is no question that interdisciplinary curricula, as called for in the “Master Plan for Medical Studies 2020”, represent progress. However, an increased amount of work in the teaching coordination of the subjects must be taken into account in the realization of such curricula in large faculties, irrespective of the efforts required to convert to a new curriculum.

## Zusammenfassung

Dieser Erfahrungsbericht vergleicht den Planungs- und Koordinationsaufwand eines Lehrkoordinators der Anatomie in einem Fächer-orientierten Regelstudiengang mit einem Fächer-übergreifenden, modular organisierten Modellstudiengang an einer Fakultät mit 600 Medizinstudierenden pro Jahr. Durch die Verteilung der anatomischen Lehre über mehrere Standorte und über Module in allen Semestern sowie Rotation dieser Module innerhalb der Semester entsteht ein erhöhter inhaltlicher Koordinationsaufwand und insbesondere ein sehr komplizierter Stundenplan. Die sinnvolle und trotzdem überschneidungsfreie Einteilung der Lehrenden der Anatomie in diesen Stundenplan wird dabei zu einer besonderen Herausforderung. Es steht außer Frage, dass Fächer-übergreifende Curricula, wie sie im „Masterplan Medizinstudium 2020“ gefordert werden, einen Fortschritt darstellen. Bei der Realisierung solcher Curricula in großen Fakultäten muss aber, auch unabhängig vom Aufwand für die Umstellung auf ein neues Curriculum, ein erhöhter Arbeitsaufwand in der Lehrkoordination der Fächer berücksichtigt werden.

## 1. Introduction

In their recent article, Maaz et al. document the transition from a classical standard curriculum to a reformed “model” curriculum at the Charité in 2009, referring to it as “moving a mountain” [1]. Like other authors [2], [3], they rightly emphasize the high workload involved in introducing a new curriculum – which sometimes made me, as the then anatomy teaching coordinator, part of the “mountain”.

But I do not want to describe curriculum reform “from the perspective of the mountain” but rather point out something that in my view is not debated enough: the permanent difference in the organizational effort, between a subject-oriented standard curriculum and an interdiscplinary modular curriculum that arises independent of the changeover-related efforts. I consider this effort to be relevant as it belongs to the “financial and capacity implications of the restructuring of the curriculum” according to the Master Plan for Medical Studies 2020 [4] – the latter is currently pending budgetary approval, as these effects have not yet been fully determined.

I did not measure this effort and at least in everyday work as a teaching coordinator it would hardly have been possible to distinguish it precisely from my other tasks and or measurable with a stopwatch. So I can only offer a personal view.

## 2. The starting point

From 2007 to 2015, I was teaching coordinator of the three institutes of anatomy of the Charité. Prior to that I had been involved in teaching organization since 2001, in particular in the Reformed Medical Curriculum (RSM) which was introduced at the Charité from 1999 for 63 students per year to run in parallel with the standard degree course [2]. My tasks as a teaching coordinator of anatomy included content coordination of anatomical teaching and the coordination of timetables with the Dean of Studies. In particular, I had to allocate about 50 colleagues to the various courses and examinations in human medicine (Standard, Reformed and Model curriculum) and dentistry. These 50 people represented a colorful mix of full- and part-time employees, outside staff, career starters, professors, doctors and non-medical professionals.

In the *standard curriculum in human medicine* (RGS) at the Charité [5], anatomical instruction was spread over four semesters. The first semester contained an introduction to general anatomy and histology, in the 2^nd^ to 4^th^ semester macroscopy and histology were taught alongside each other and were thematically coordinated with each other and the subjects of physiology and biochemistry (musculoskeletal system, internal organs, head/neck, CNS/senses). Lectures, seminars and dissection and microscopy courses each ran through the semester on the same weekdays. Accompanying oral and written examinations (as a prerequisite for a course certificate) were organized by the institute alone, the final “Physikum” together with the state examination office.

The "Modellstudiengang Medizin" (*model curriculum medicine*, MSM) which was introduced at the Charité [1], [6] for 300 students per semester in the winter semester 2010/2011 is organized across all disciplines and in a completely modular way. Only its first implementation version is described here in a simplified form (cf the module plan on page 474 of the Study Regulations [6]). Each semester consists of four thematically delimited 4-week modules, which are usually offered twice per semester for one half of the cohort (to distribute teaching over more than four weeks for the clinics involved in each module). Some modules are also offered only once or three times. In any case, students do not all complete the modules in the same order. In the first few semesters modules are predominantly on organ systems (e.g. cardiovascular, respiratory, sensory) while diseases predominate in higher semesters (e.g. abdominal disorders, neurological disorders). Anatomy is part of modules from the 1st to 9th semesters, with about three-quarters of the anatomical teaching spread over the first four semesters. This includes a dissection course in which groups dissect a donor body over the 3rd and 4th semester, largely in line with the modules. Seminars mainly take place as interdisciplinary seminars, mostly with clinicians. Overall, the number of subject-specific teaching hours is less than in the RGS and instead anatomy teaching staff are involved in general teaching formats such as PBL or communication training. Instead of course-related examinations there are centrally organized semester final examinations. The only purely anatomical examination is the “3D-MC” which has been carried over from the RSM. It consists of a tag test at the end of the 3rd Semester [7].

Subsequent modifications to the original MSM curriculum, which amongst other things included shortening of some modules and an increase in the number of lecture hours (see the module plan on page 1353 of the 2015 Study Regulations [8]) are not considered here but did not alter the fundamental differences between the standard and model curriculum.

## 3. Comparison of effort

At first, the new curriculum also led to a big reduction in the workload of the teaching coordinator: Awarding course certificates and the corresponding organization and documentation of course-accompanying exams (four MC exams, six oral exams) fell by the wayside. At some point, the weekly consultation I offered in the RGS was no longer being used because negotiations with individual students about equivalencies, retaking exams, recognition of absences, etc. went to newly founded “module secretariats” (which also meant that I was less and less involved with the fate of individual students). However, as a result the anatomy teaching secretary became a module secretary and was no longer available to me.

In terms of exam organization, the overall effort more or less remained the same: the “Physikum” and course exams disappeared and instead, anatomy was part of the end of semester exams and we had to organize the 3D-MC, which, however, represents a major logistical challenge with 300 examinees.

**Figure 1:**
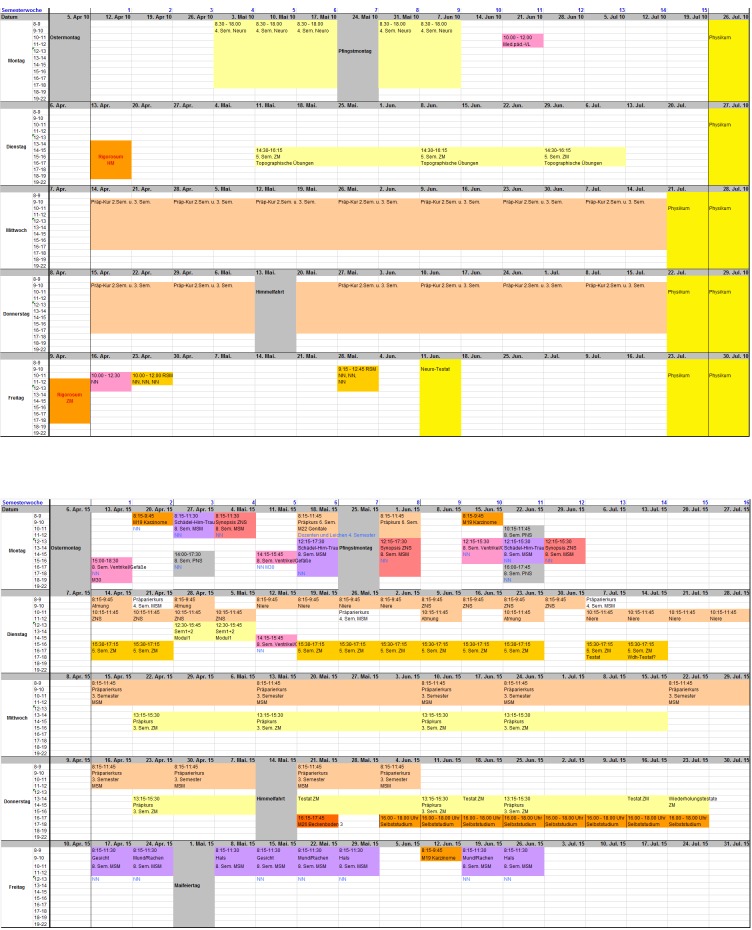
Use of the same dissecting room in the summer semester 2010 (above) and in the summer semester 2015 (below). The 5 large lines represent the weekdays Monday to Friday, each divided into time slots from 8AM to 7PM, the columns represent 16 semester weeks (2010 in week 15/16: Occupancy by the state exam “Physikum”). Names have been replaced by “NN”. In 2010, the room was allocated to continuous dissection courses on Wednesdays and Thursdays, a neuroanatomy course on Mondays, and a course for dental students on Tuesdays. In 2015 courses for dental students occupy Tuesday, Wednesday and Thursday afternoons. All other courses in various colours are MSM courses in semesters 1-8, including a continuous dissection course on Wednesday and Thursday mornings.

Planning and allocation of anatomical teaching staff in the MSM was characterized by “new complexity” in the MSM (see figure 1 (Fig. 1)) as this now had to be distributed over nine semesters and had to be coordinated not only with the associated disciplines of the pre-clinical phase but with all participating disciplines in the modules. This new complexity is also reflected in the growth of the Study Regulations from 10 to 53 printed pages [5], [6]. The anatomy department was involved in 28 modules from the 1st to 9th semester The modules rotated in different patterns from semester to semester and were offered once, twice or three times in the four possible 4-week slots. Thus, for example, the module “Growth, Tissue, Organ” of the 2^nd^ semester took place only in the 1^st^ slot, the module “Skin” of the 3^rd^ semester in the 1^st^ and 2^nd^ slot, the module “Kidney” of the 4^th^ semester in the 2^nd^ and 4^th^ slot and the module “Sexuality” of the 6^th^ semester in the 2^nd^ and 3^rd^ slot. Within modules, anatomical teaching was not always evenly distributed over the weeks either, as this distribution was primarily dependent on content-related factors. The resulting, extremely complicated overall timetable for anatomy thus changed from week to week – it is no coincidence that one feature of reformed degree courses is that staff have to be reminded of each of their teaching sessions by email.

The faculty managed central timetable planning by developing its own teaching database [9]. However, this did not free the teaching coordinator from having to reconcile the content of anatomical teaching and to allocate teaching staff appropriately without overlaps. This was done using Excel^®^ tables we created since the central database did not permit trial filling and moving of teaching allocations. In addition, the increased proportion of interdisciplinary teaching inevitably led to anatomy no longer being taught completely at the anatomical institute, so that in some cases planning times had to take journey times between locations into account. Another complicating factor was that in the transition from RGS to MSM, overall teaching time (contact time with students) for lecturers increased by over 40%, as can be calculated from the published Study Regulations [5], [6]. This increased the likelihood of overlaps and occasionally caused frustrations amongst the teaching staff, who now had to try to reconcile their own research with increased teaching contact hours in unpredictable patterns and in changing locations.

As mentioned above, I have not been able to measure my own increase of coordination efforts but can illustrate this using planning documents: In the standard degree course, it was possible to disseminate the basic allocation of teaching staff to the various courses in a 6 to 7-page Word^®^ document to colleagues (including RSM and dentistry) because for a dissection course in the 3rd semester for example, the weekdays were fixed, so that an allocation of a teacher to a group of students and a room was sufficient for the whole semester. In addition, each of the four semesters had a two-page lecture schedule, which announced the lecture topics and courses and allocated the lecturers. The teaching allocation of an entire semester could therefore be contained on some 15 A4 pages. The teaching effort in weekly semester hours (SWS) per person, which had to be based on the mandated individual teaching load and was important for an acceptable fair distribution of teaching, was relatively easy to estimate “manually” (e.g. a dissection course of the 3^rd^ semester being equivalent to 4 SWS).

In the MSM, the basic overview, with which I “explained” the teaching content to all my colleagues, grew to about 20 pages. The actual allocation was now in an additional Excel spreadsheet, which was filled with over 3,000 individual events and which the various drafts and final versions had to be run by the teaching staff before the final allocation was entered in the above-mentioned central faculty database. Using automated calculations in Excel, it was possible to detect planning overlaps and to consider the individual teaching effort in SWS. Since this could no longer be estimated at a glance, it had to be calculated from the sum of all assigned individual events.

## 4. Conclusion

For me, there is no question that we need curricula in which communication and practical relevance play a greater role, didactically reduce specialist knowledge and make subjects more interlinked. Interdisciplinary teaching is, for example, not only prescribed by the Medical Licensure Act but is also fun and instructive for the teaching staff. All I have shown here is that in my case all of this was associated with a massive increase in the amount of work involved in the coordination of teaching anatomy, regardless of the extra work required to introduce a new curriculum, i.e. irrespective of any inherent change management required. Whether this also applies to other subjects or other model courses I leave to my colleagues to report. I have also not tested whether available scheduling software can really help with the complex tasks described.

Based on my own experience to date, it is still possible to keep an overview over your subject as long as student numbers are relatively low, such as the 63 per year in the Charité reformed curriculum [2] or 48 per year in the model curriculum at the Brandenburg Medical School [10], even in a modular interdisciplinary system. However, with the 600 students per year at the Charité, this overview was completely lost so that I was more concerned with Excel spreadsheets than with the teaching quality. This raises the question of whether the above-mentioned goals are really only achievable with a modular structure and the associated complicated timetable. After all, the word “module” did not appear in the master plan [4] although the most recent recommendations of the expert commission on the master plan refer to modularization as an “essential structural principle” [11].

However, even without modules, additional costs for subject coordination will not be avoidable if the Master Plan 2020 is to be realized, since the coordination of interdisciplinary teaching is more time-consuming than the organization of subject courses. This was also recently confirmed by the expert commission [11], which, however, remained vague in quantifying this additional expense. Maybe the implementation of the master plan could be combined with a reduction of the teaching load for coordinators? That would not be my personal goal as I enjoy teaching but it would at least substantiate the extra effort. This extra effort has to be taken into account somehow, so that the subject teaching coordinators do not become part of a mountain that does not want to move.

## Competing interests

The author declares that he has no competing interests.
